# Reasons, Years and Frequency of Yoga Practice: Effect on Emotion Response Reactivity

**DOI:** 10.3389/fnhum.2018.00264

**Published:** 2018-07-04

**Authors:** Elisabeth Mocanu, Christine Mohr, Niloufar Pouyan, Simon Thuillard, Elise S. Dan-Glauser

**Affiliations:** Institute of Psychology, University of Lausanne, Lausanne, Switzerland

**Keywords:** yoga, emotion reactivity, regulatory behavior, emotional experience, cardiovascular response, respiratory response

## Abstract

Yoga practice, even in the short term, is supposed to enhance wellbeing and counteract psychopathology through modification of emotion reactivity. Yoga teaches that emotional responses may be less pronounced with longer and more frequent practice, and potentially when people perform yoga for mental rather than physical reasons. We tested 36 yoga practitioners of varying experience (between 6 months and 11 years of practice). We assessed participants’ self-reported emotional experience and peripheral physiological reactions (heart rate, skin conductance, respiration) when seeing positive and negative pictures. Results were analyzed as a function of the years of, frequency of, and reasons for yoga practice. We found a heart rate increase with the degree participants performed yoga for mental reasons. In addition, years of yoga practice were significantly associated with reduced abdominal respiratory rate when facing negative pictures, speaking in favor of reduced arousal with yoga experience. Finally, regarding frequency of practice, a higher frequency in the last month was linked to less negative and positive experiences as well as a reduced abdominal respiratory amplitude when viewing positive pictures. Altogether, these results demonstrate that intense short-term yoga practice might relate to a (i) decrease in the intensity of self-reported emotional experiences and (ii) deepened respiration. Short-term effects might be shaped by what participants expect as practice benefits. However, several years of practice might be needed to decrease respiratory arousal in the face of negative situations, which likely is a manifestation of an evolution in the emotion regulation process.

## Introduction

According to pictures and manuscripts, yoga was initially practiced in various regions across India, and most likely originated from the culture of *Greater Magadha* (Bronkhorst, 2007). The term yoga as a spiritual practice appears around year 0 but the Yoga Sutras, and their principal commentaries, the Yoga Bhasya, are considered as being the first written texts describing the method of Yoga. Probably written by *Patanjali*, these written texts are actually relatively recent (circa 400 A.D., Bronkhorst, 1985). Massively influenced by Buddhism thereafter, it was brought to the United States in 1893 and later to Europa through Swami Vivekananda, a Bengali saint spreading ‘bhakti yoga’ (Williams, 1995, 2001; Hoyez, 2007).

It is challenging to summarize the essence of such an old tradition, if not philosophy, in a couple of sentences. We lend for this purpose what Gard et al. (2014) stated: “yoga, originating from India, is an ancient contemplative practice dating back over 3500 years, which aims at one thing – to alleviate suffering and promote optimal physical and mental thriving” (page 2). In the *Raja yoga* tradition, this can be achieved through practicing the so-called eight « limbs », representing universal moral and ethical principles, individual self-restraint, physical postures, breath control, calming of the senses, concentration, meditation, and pure contemplation (Gard et al., 2014). Thus, these limbs tap on both mental and physical processes, and, by inference, shape a coordinated lifestyle.

In recent years, studies have reported that yoga is both beneficial for wellbeing and a potent clinical intervention, e.g., for stress reduction (Chong et al., 2011; Muehsam et al., 2017). In addition, yoga is considered to alleviate numerous psychiatric and physical conditions such as schizophrenia, depression, anxiety, and posttraumatic stress disorder (PTSD) (Cabral et al., 2011; Hofmann et al., 2016; Klatte et al., 2016), as well as metabolic syndrome (Cramer et al., 2016), menopausal syndrome (Cramer et al., 2012), diabetes mellitus (Cui et al., 2017), chronic heart failure (Gomes-Neto et al., 2014), and obesity (Lauche et al., 2016).

Beyond yoga’s promise as an intervention, yoga is interesting to psychology in general. Yoga and related Eastern traditions complement and mirror many assumptions found in Western psychological traditions (Caplan et al., 2013). Yoga practice aims, for instance, to minimize mind wandering, which could be compared to enhanced focused attention (Braboszcz et al., 2017) as well as reduced rumination (Kinser et al., 2013; Schuver and Lewis, 2016). Many contemporary cognitive-behavioral treatments focus on using top**–**down cognitive means of self-regulation, such as cognitive reappraisal, reframing, and goal-setting (DeRubeis et al., 1990; Garratt et al., 2007; Berking et al., 2008). Gard et al. (2014) found that age-associated decline in fluid intelligence was counteracted and functional brain networks more efficient in long-term yoga practitioners as compared to matched controls. Finally, with more yoga practice, individuals showed less dysfunctional coping styles, enhanced emotional mental and physical disengagement when asked to change appraisal of affective meaning of aversive pictures (Dale et al., 2011). All in all, results uncover the profound impact of yoga practice on the organism, both at structural and functional level. These observations go well with Caplan et al. (2013) proclaiming that integration of yogic wisdom and psychological knowledge enriches our understanding of recent insights in psychology as well as interventions in the health and psychopathological domains.

The observations on yoga so far are unquestionably relevant to psychology. Unfortunately, such observations do not explain the mechanisms through which any positive impact arises, nor does it prove that any positive impact originates from yoga practice *per se*. Without claiming to be exhaustive, we here detail three points, which, in our view, deserve closer discussion: emotional reactivity and its relation to relaxation, the placebo effect, and the years of practice versus quality of training regarding effects of yoga practice.

Under emotional reactivity, we can understand the extent to which people experience emotional reactions (Nock et al., 2008), which involve changes in experience, expression, and physiological arousal (Izard, 1977; Buck, 1984; Levenson, 1994, 2000). Emotions can be caused by internal events such as thoughts or memories, or by specific external events (Frijda, 1986). The way we react to the latter stimulation (environmental situations) is highly relevant to well-being and psychopathological development. Concerning well-being, emotional stability trait, i.e., the avoidance of mood swings of high amplitude, leads to increased life-satisfaction (Diener et al., 1999; Vittersø, 2001). Also, moment-to-moment rise and intensity stability in emotional reactions are associated with greater well-being, as measured by reported absence of clinical symptoms, greater life satisfaction, or higher frequency of positive emotions (Houben et al., 2015). Concerning psychopathology, strong autonomic changes in the face of stressors experienced in early childhood relate to present (Boyce et al., 2001) and prospective (Pine et al., 2014) psychopathological symptoms. For example, greater negative emotional reactivity to disaster-related images after the earthquake tsunami, and nuclear crisis in Japan in 2013 predicted higher depressive and post-traumatic symptoms (Cavanagh et al., 2014). Pruneti et al. (2011) showed that skin conductance responses were more frequent in anxiety disorder than controls, but these were less frequent in patients with depression and anorexia than controls. These last results point to associations between emotional reactivity and psychopathology that involve both hyper- and hypo-reactivity, psychopathology being associated with both increased as well as blunted emotional reactions (see also D’Andrea and Pole, 2011).

To return to yoga in the context of emotional reactivity, some yoga limbs relate independently to emotional reactivity and regulation as well as to respective clinical interventions. When considering the limb of physical postures, taking particular body postures influenced individuals’ emotional state, likely via proprioceptive feedback, i.e., individuals feel the emotion that corresponds to the adopted posture (Duclos et al., 1989). Training relaxation or cognitive coping skills helped students in the short and long term (1 year later) to develop superior anger management skills (Hazaleus and Deffenbacher, 1986). Also, long-term mindfulness meditation has been shown to increase emotional stability (Taylor et al., 2011). Yoga components were also implemented in established clinical interventions. For instance, cognitive-behavioral interventions aim to enhance patients’ self-regulation abilities with cognitive reframing (e.g., goal setting) as well as acceptance and tolerance (e.g., Berking et al., 2008). In patients with PTSD due to repeated childhood abuses, mindfulness-based intervention of hatha yoga presented a well-perceived complementary treatment (West et al., 2017). This intervention did not only focus on symptom reduction, but also on personal growth. Patients reporting on their treatment experiences identified five major themes, i.e., participants’ feelings of gratitude and compassion, relatedness, acceptance, centeredness, and empowerment. Thus, emotion reactivity and regulation might represent important mechanisms that could explain the positive impact of yoga practice.

Concerning the second point, i.e., the placebo effect, it may explain the positive impact of yoga practice, or represent a confound. Some authors argue that the same positive consequences as that of yoga practice could be achieved through other types of physical exercise (Ross and Thomas, 2010). Also, simply entering a mode of activity may provide increased self-esteem, improved mood, and reduced anxiety (Fox, 1999). It is a challenge to select the “right” control group when being able to select amongst numerous options. For example, the control group might involve other sorts of treatments, such as cognitive behavioural therapy (CBT) (Boehm et al., 2012) or being simply on a waiting list (Javnbakht et al., 2009; Bussing et al., 2012). Such differences in control groups may lead to different results. Most important here, such study protocols cannot prove that the positive impact of yoga practice originates from yoga itself (reinforcing the “placebo” argument). Researchers discuss appropriate control conditions that commonly involve some form of exercise such as walking, dancing, swimming and other sorts of aerobic exercise (Berger and Owen, 1988, 1992), but can also involve meditation (Auty et al., 2017) or concentration on reading a newspaper (Schell et al., 1993). When taking into account the findings of these more appropriately controlled studies, research shows that yoga is as efficient as exercise if not more desirable in its effects (Ross and Thomas, 2010).

Finally, we further extend on our questions of genuine yoga effects by highlighting the possible importance of the years of practice, quality of, and reason for yoga training. Often, individuals participate in yoga training of relatively limited and a fixed number of intervention sessions (mostly about 2 sessions per week for about 6–12 weeks, see Streeter et al., 2010; Field et al., 2013; Davis et al., 2015; Amaranath et al., 2016; de Manincor et al., 2016). Measures of interest (e.g., anxiety, depression level) are assessed before and after this training. Duration and depth of exercise as well as reason and motivation to exercise might, however, differ. Unexperienced individuals are likely to use more top-down cognitive processes and experienced individuals more bottom–up processes in their self- and emotion-regulation processes (Chiesa et al., 2013; Gard et al., 2014). In the same vein, unexperienced yoga practitioners may rely on physical reason for performing yoga, whereas experienced practitioners could see yoga as a more spiritual practice (Park et al., 2016). Putting wisdom and experience of ancient, cultural traditions in a new cultural context of 8–12 weeks training is possible and done, but whether this approach guarantees that desirable effects are achieved within such short periods of time needs to be established (Alexander et al., 2008; Aljasir et al., 2010). In our view, nobody would claim that a musical instrument, gymnastics or a ball game could be mastered in such a limited period of time and number of sessions. The choice of design permitting to highlight yoga effects of genuinely performed, self-motivated, and potentially long-term practice is thus of importance.

Taking into consideration above observations and questions on genuine yoga effects, we here investigated healthy participants’ emotional reactivity as a function of years and intensity of yoga practice as well as reasons to perform yoga. Participants were exposed to positive and negative pictures. We assessed responses to these pictures by asking participants to rate their emotional experience and by recording their heart rate, respiration, and skin conductance. Participants reported on their reason to practice yoga (mental and / or physical benefits), when they started practicing yoga, how often they practice yoga in general (in hour per week) and practiced yoga recently (hours/week over the last month). Thus, we were able to dissociate short-term training effects and long-term expertise as well as frequency effects. We expected attenuated self-reported emotional intensity ratings for both picture types (Desbordes et al., 2015) with more and longer yoga training, in particular when performing yoga for mental reasons. Regarding physiological reactivity, we expected reduced electrodermal reactions with a higher number of years and frequency of yoga practice, as well as a steadier respiration, particularly for negative stimuli.

## Materials and Methods

### Participants

Thirty-six volunteers (11 males and 25 females) were recruited via advertisements in two yoga schools and posted on the internet. Their ages (always in years) ranged from 21 to 56 (mean age 30.7; standard deviation, SD, 8.2). Education levels of the participants included University degrees (18 with a Master degree or higher and 10 with a Bachelor), professional diplomas (*n* = 5), and lower education (secondary diploma; *n* = 4). Inclusion criteria were general good health, good understanding of French, and being currently involved in some kind of yoga practice. Twenty-three participants reported performing one practice type (14 Hatha, 8 Power Vinyasa, 1 Ashtanga). Ten participants reported to combine two yoga branches, six of which were focused on Power Vinyasa and Hatha. Finally, one last participant reported three kinds of practice (Power Vinyasa, Hatha, and Ashtanga). Two participants declined to answer regarding their practice type.

### Emotion Reactivity Task

We selected 55 pictures from the Geneva Affective PicturE Database (GAPED, Dan-Glauser and Scherer, 2011). The GAPED gathers negative and positive stimuli that were pre-rated on valence and arousal in a similar population. For the current purpose, we selected 20 negative and 20 positive pictures. We added 15 neutral pictures for diluting the effect of opposed positive vs. negative contents, as well as to include non-emotional content (they were not further considered in the analysis). Based on ratings furnished with the GAPED, negative pictures had a mean valence rating of 31.16 (0 being extremely negative and 50 being neutral, *SD* = 8.38) and positive pictures had a mean valence rating of 79.59 (50 being neutral and 100 being extremely positive, *SD* = 4.94). These valence ratings differed between picture categories, *t*(38) = –22.26, *p* < 0.001. Regarding arousal, negative pictures had a mean arousal rating of 47.65 (0 being not arousing and 100 extremely arousing, *SD* = 8.91). On the same scale, positive pictures had a mean arousal rating of 25.81 (*SD* = 12.80). In line with previous reports (Garavan et al., 2001; Grühn and Scheibe, 2008), arousal is higher for negative than positive pictures, *t*(38) = 6.26, *p* < 0.001.

During the actual task, participants were seated in front of a computer screen at a distance of approximatively 60 cm. We presented the 55 pictures (each for 8 s) in a randomized order in the center of the screen. Pictures had a size of approximately 26 × 22 cm. Participants were asked to carefully look at the pictures, and to report their emotional feeling via a response box consisting in a slider (see also next section). After each picture presentation, they were prompted (written information on computer screen) to reset the slider to the center before the next trial automatically commenced. Duration of the entire trial (picture and inter-picture interval) was 11 s.

### Emotion Reactivity Measures

We assessed participants’ emotional experience by using a response box consisting in a slider that allows participants to continuously rate their emotion experience (Variable Assessment Transducer, Biopac Systems, Inc., Goleta, CA, United States). Thus, we could assess the subjective emotional experience across time, i.e., during the whole picture presentation. Participants used their right hand to move the slider according to their felt emotion intensity along a 11 cm line. The slider was anchored on the left side with *very negative* and on the right side with *very positive*.

We assessed participants’ physiological reactions to the pictures with three autonomic responses. The first autonomic response was electrocardiography (ECG), measured with three standard disposable, pre-gelled Ag/AgCl electrodes disposed in one of the Einthoven III configuration. One electrode was placed approximately 5 cm below the lower rib on the left side of the abdomen. A second electrode was placed just under the right clavicle, along the mid-clavicular line. A third electrode, which functioned as a ground, was placed at the level of the xiphoid process. The second autonomic measure was electrodermal activity. The skin conductance level was recorded with two pre-gelled disposable Ag/AgCl sensors from Biopac Systems (Goleta, CA, United States). They were placed on the thenar and hypothenar eminences of the left hand palm. The third autonomic measure, respiration, was recorded on two different sites, thoracic and abdominal. While both measure respiration, these measures target the activity of different muscles involved in breathing. Having these two measures is particularly relevant with respect to yoga, a practice that frequently focuses on modifying breathing dynamics. Indeed, it is suggested that recruitment of lower muscles, involved in so-called diaphragmatic breathing, may be more adaptive as this kind of breathing reduces muscle constraints and increases ventilation efficiency while decreasing anxiety (Shaw and Shaw, 2011). This is why in yoga practice, and especially Pranamaya practice, various breathing exercises are taught with the aim of restoring diaphragmatic breathing (Goyeche et al., 1982). Respiration recordings were gathered with two respiration belts from Biopac Systems (Goleta, CA, United States). The abdominal belt was placed around the waist just above the pants, whereas the thoracic belt was placed high on the chest just below the armpits. All emotion parameters were recorded and amplified with MP150 compatible modules from Biopac Systems (Goleta, CA, United States). All channels were sampled at 1000 Hz.

### Self-Report Measures

#### The Difficulties in Emotion Regulation Scale (DERS, Gratz and Roemer, 2004)

The DERS is a 36-item questionnaire assessing difficulties in emotion regulation. It evaluates, among other sub-scales, to what extent the responder lacks emotional awareness and has difficulties in emotion identification. The two subscales are particularly relevant when emotion response involve self-reported emotional feelings. Items include sentences such as “I am clear about my feelings” and “I have difficulty making sense out of my feelings.” The participant has to indicate on a five point Likert scale from 1 = almost never to 5 = almost always, the frequency of the behavior described in each item. We use the French version of the questionnaire DERS-F (Dan-Glauser and Scherer, 2013).

#### Reasons for Yoga Practice

Originally, we had planned to test two groups of people as a function of their reason for yoga practice. We had in mind separating groups according to whether they practiced yoga for physical or mental reasons. In order to know their prevalence, we performed a pilot study at a local yoga school. We asked 24 participants (14 women, 6 men, 4 did not report their gender) with a mean age of 37.55 years (*SD* = 8.74, ranging from 21 to 55) on reasons for yoga practice and on their yoga experience so far (e.g., how often they practice yoga since they started). Interestingly, individuals early in their yoga practice (several weeks to months) could largely be differentiated according to these pre-defined groups (mental vs. physical reason). Some practiced yoga predominantly for physical health benefits (e.g., flexibility of the body, back pain). Others practiced yoga predominantly for mental reasons including enhanced concentration capacities, meditation aspects, and better clarity of mind during or after practice. Yet, when we considered responses of individuals who practiced yoga for several years, the two groups (mental vs. physical reasons) could not be differentiated anymore: individuals with longer experience seemed to practice yoga for both physical and mental reasons as their practice motive as Park et al. (2016) have also found. Individuals indicated that they perform yoga for both physical and mental reasons. Consequently, the idea of grouping participants based on their practice intention was no longer justified. Instead, we asked participants about the extent to which they practice yoga for physical (“To what extent are you performing yoga for physical reasons?”) and mental (“To what extent are you performing yoga for mental reasons?”) reasons. Answers to these two questions were gathered on two analog scales ranging from 0 (not at all) to 100 (totally).

#### Years and Frequency of Yoga Practice

We asked participants on number of years and frequency of practice with the questions: (a) “For how long (in years) have you been practicing yoga?” (Years of practice); (b) “How many hours per week, on average, have you been practicing yoga?” (General frequency), and (c) “How many hours per week have you been practicing yoga in the month prior testing?” (Recent frequency).

### Overall Procedure

Participants were welcomed to the lab and informed about the procedure. After signing the informed consent form, they were asked to complete the questions about frequency and years of yoga practice. Participants were then prepared for the physiological recordings and had to answer questions about their health status. We made sure all their questions about the settings were answered and signals from all channels were checked. The DERS-F was completed either before or after the main task, in randomized order. Participants were then left alone; all instructions being presented on screen. In the first part, participants answered the two questions about the degree of physical and mental reasons for which they practice yoga. Participants were then presented with the rating slider and explained that the task of the study was to view a certain number of pictures and report their feelings according to these pictures by moving the cursor. A few training trials were then proposed in order for them to get accustomed to the task. After the computer session, which lasted about 20 min, sensors were removed and participants were fully debriefed.

### Data Extraction

Regarding the questionnaires, subscale scores of the DERS about (a) lack of emotional awareness and (b) emotional clarity were extracted for control purpose. Scores for the questions regarding the degree of physical and mental reason were used as given (score ranging from 0 to 100). Number of years of practice (year) and frequency (hour per week for general frequency, and hour per week over the last month for recent frequency) were extracted as indicated. All the experience and physiological recordings were treated with Acknowledge 4.4 (Biopac Systems, Goleta, CA, United States). All channels were band-pass filtered to increase signal to noise ratios. Channels were then manually scanned for remaining artifacts, which were corrected via signal interpolation or excluded. In addition to the picture presentation period (8 s), a baseline of 3 s was calculated for each trial. This period spanned from 3 s before the picture presentation to the time of the picture onset. Regarding emotional experience, ratings were exported to obtain mean values for positive and negative pictures. Rating data were then transformed into two scales (depending on the valence) from 0 = absence of emotional feeling to 100 = extreme negative or positive emotion. Regarding autonomic responses, heart rate was calculated from the ECG channel by transforming the inter-beat interval (i.e., the duration between successive R waves). Skin conductance level was exported as mean values for each stimulus. Respiratory rate and respiratory amplitude were calculated for each stimulus. The respiratory rate was obtained by converting the duration of the cycle intervals into a number of cycles per minute (c/min). The respiratory amplitude was interpolated by using the difference in volts between the point of maximum inspiration and the point of maximum expiration. All these response channel data were calculated as the change in activity with respect to each trial baseline.

### Data Analyses

Previous research showed different emotion and emotion regulation patterns for positive and negative emotions (Hubert and de Jong-Meyer, 1991). Thus, we performed separate analyses for negative and positive trials. For each independent and dependent parameter, outlier participants (> or < to 3 inter-quartiles) were removed for analysis. Thus, one participant was excluded for the variable *years of practice*, two for *frequency of recent yoga practice*, six for *skin conductance level* (which included five non-responders for this channel), two for *respiratory amplitude for thoracic site-negative* and for *abdominal site-positive*, and five for *abdominal respiratory amplitude for negative stimuli*. We performed separate stepwise linear regressions to test our hypotheses, each time evaluating how our yoga practice parameters would predict participants’ emotion reactivity responses in the context of negative and positive stimulations. Outcome variables were either the self-reported experience of the emotion or the physiological reactions to the pictures, measured with heart rate, skin conductance, thoracic and abdominal respiratory rate and amplitude. For each regression, we included the following predictor variables (a) reason for yoga practice (mental and physical), (b) years of yoga practice, as well as (c) general and recent frequency of yoga practice. We found several significant Pearson’s correlations between age and other variables (see below under participants). Consequently, we added age to the first block of all stepwise, hierarchical regression analyses. We report the details of the significant results.

*P-values* were not corrected for multiple comparisons across our regression analyses, following the rationale described in Rothman (1990) and Perneger (1998). Indeed, the correction for multiple comparisons aims to control Type I error, but overly inflates Type II error. We are here performing the first study of its kind. We designed this study to explore elements of yoga practice that could influence emotion reactivity. Our approach here is to help identify variables that are worth being investigated in future studies and might help confirming or describing mechanisms at play.

## Results

### Participants

Participants had equally strong reasons to practice yoga for physical and mental reasons (**Table 1**). We observed a wide range of number of years of yoga practice ranging from under a year to about 11 years (**Table 1**). On average, participants practiced over 2 h per week in general and did so over the last month (**Table 1**). We found that increasing age was associated with (i) diminished levels of practicing yoga for mental reasons (*r* = –0.38, *p* = 0.022), (ii) practicing yoga over a longer period of time (*r* = 0.39, *p* = .020), and (iii) practicing yoga more frequently over the last month (*r* = 0.41, *p* = 0.017). Age was unrelated to levels of practicing yoga for physical reasons (*r* = –0.20, *p* = 0.23), overall frequency of practice (*r* = 0.28, *p* = 0.09), or any of the emotion reactivity responses (*r* = –0.24–0.14, *p >* 0.16).

**Table 1:** Mean, SEM, 95% CI, and range of reasons, years and frequency of yoga practice, as well as of emotional responses for negative and positive pictures.

		Mean	SEM	CI	Range	Mean comparison
Age		30.71	1.37	[21.92; 33.50]	21.29–55.97	
DERS		2.16	0.11	[1.94; 2.37]	1.31–4.44	
Physical reason		67.47	2.97	[61.44; 73.50]	20.24–100	
Mental reason		69.04	3.32	[62.30; 75.78]	29.78–100	*t*_(35)_ = -0.41, *p* = 0.68
Years of yoga practice		3.7	0.48	[2.72; 4.68]	0.5–11	
Frequency (hour per week)		2.51	0.28	[1.94; 3.08]	0.25–6	
Frequency over the last month (hour per week)		2.39	0.41	[1.56; 3.23]	0–10	
Experience	Negative	20.36	2.55	[14.84; 26.19]	-10.51–51.07	
	Positive	23.78	1.80	[18.72; 26.57]	-2.42–42.00	*t*_(35)_ = -1.44, *p* = 0.16
HR	Negative	0.69	0.20	[0.20; 1.11]	-1.82–3.19	
	Positive	0.33	0.15	[0.01; 0.69]	-2.23–2.47	*t*_(35)_ = 1.68, *p* = 0.10
SCL	Negative	0.12	0.04	[0.04; 0.2]	-0.11–0.56	
	Positive	0.02	0.02	[-0.06; 0.04]	-0.33–0.40	*t*_(2)_ = 2.33, *p* = 0.03^∗^
Thoracic RR	Negative	0.04	0.08	[-0.15; 0.19]	-1.05–1.18	
	Positive	0.09	0.09	[-0.03; 0.36]	–0.85–1.79	*t*_(35)_ = -0.50, *p* = 0.62
Thoracic RA	Negative	28.65	25.26	[-20.65; 109.18]	-299.43–383.29	
	Positive	-30.00	33.90	[-122.18; 36.18]	-648.31–498.90	*t*_(33)_ = 1.26, *p* = 0.22
Abdominal RR	Negative	0.00	0.09	[-0.20; 0.17]	-0.83–1.60	
	Positive	-0.03	0.10	[-0.25; 0.23]	-1.48–1.43	*t*_(35)_ = 0.27, *p* = 0.79
Abdominal RA	Negative	-1.67	8.06	[-23.57; 18.58]	-167.55–83.10	
	Positive	-8.36	7.47	[-38.53; -2.14]	-148.40–66.8	*t*_(30)_ = 1.38, *p* = 0.18


*Physical and Mental reason scales are from 0 (not at all) to 100 (totally). The experience scale goes from 0 (no emotion) to +100 (very negative/positive). All other parameters are differences from baseline level. HR, Heart Rate in bpm; SCL, Skin Conductance Level in μS; RR, Respiratory Rate in c/min; RA, Respiratory Amplitude in mV. The mean comparison column reports the t-test results evaluating the mean difference between performing yoga for mental vs. physical reasons and between the viewing of positive and negative pictures for the emotional response section. ^∗^p < 0.05.*

Distribution of data were similar for participants with higher education and those with lower education for all variables (Mann–Whitney *U* tests, *U-values* between 59 and 106, *p-values* between 0.135 and 0.872). Regarding DERS-F scores, our sample showed no particular difficulty in emotion regulation, the mean score was 2.15 (*SD* = 0.53). Regarding the two subscales we wanted to control, participants reported low difficulties in emotional awareness (2.14, *SD* = 0.51) or identification (1.98, *SD* = 0.70).

### Emotional Reactivity Measures for Negative and Positive Pictures

Comparing the dependent measures (experience and physiological reactions) between positive and negative picture presentations, we observed that the self-reported emotional intensities were comparable between picture types (**Table 1**). Likewise, the two picture types did not differ regarding heart rate, thoracic and abdominal respiratory rate and amplitude, respectively (**Table 1**). Skin conductance levels were higher for negative than positive pictures (**Table 1**).

### The Impact of Yoga Practice on Emotion Reactivity Measures

#### Self-Reported Emotional Experience

**FIGURE 1:**
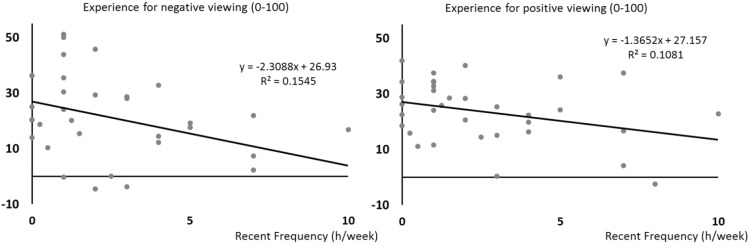
Scatterplots of self-reported emotional experiences (*y*-axis) when seeing negative pictures (left) and positive pictures (right) as a function of the recent frequency of yoga practice (hours per week over the last month, *x*-axis). Regression lines, equations, and *R*^2^ for each univariate model are shown.

We found that the more individuals practiced yoga recently, the less they reported the pictures to be emotional. This relationship was found for negative pictures, *F*_(1,31)_ = 6.87, *p* = 0.013, *R*^2^= 0.181, β = –0.43, and positive pictures, *F*_(1,31)_ = 4.95, *p* = 0.034, *R*^2^= 0.138, β = –0.37 (**Figure 1**).

#### Cardiac Emotional Reactivity

**FIGURE 2:**
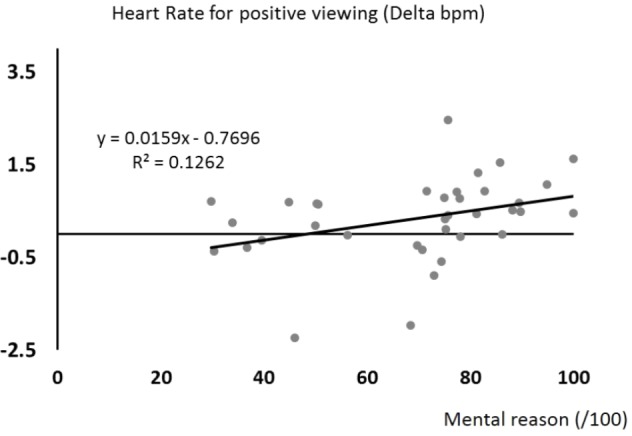
Scatterplot of heart rate changes (*y*-axis) when participants were confronted to positive pictures as a function of the extent to which participants performed yoga for mental reasons (*x*-axis). The regression line, equation, and *R*^2^ for this univariate model are shown.

None of the tested variables yielded a significant impact on the heart rate change when seeing negative pictures, *F*_(5,27)_ = 1.24, *p* = 0.32. However, the more participants practiced yoga for mental reasons, the stronger was their heart rate increase when seeing positive pictures, *F*_(1,31)_ = 5.60, *p* = 0.024, *R*^2^= 0.153, β = 0.39 (**Figure 2**).

#### Exocrine Emotional Reactivity

None of the models examining effect of yoga practice on skin conductance levels yielded significant results. This was the case for negative viewing, *F*_(5,22)_ = 0.45, *p* = 0.81, and for positive viewing, *F*_(5,21)_ = 0.33, *p* = 0.89.

#### Respiratory Emotional Reactivity

We examined respiration at two sites, the thoracic and the abdominal regions. The regression models evaluating thoracic respiration yielded no significant results, neither for the respiration rate parameter, *F*_(5,27)_ = 0.40, *p* = 0.84 (negative viewing) and *F*_(5,27)_ = 1.10, *p* = 0.38 (positive viewing), nor for the respiration amplitude parameter, *F*_(5,26)_ = 1.05, *p* = 0.41 (negative viewing) and *F*_(5,27)_ = 1.36, *p* = 0.27 (positive viewing).

**FIGURE 3:**
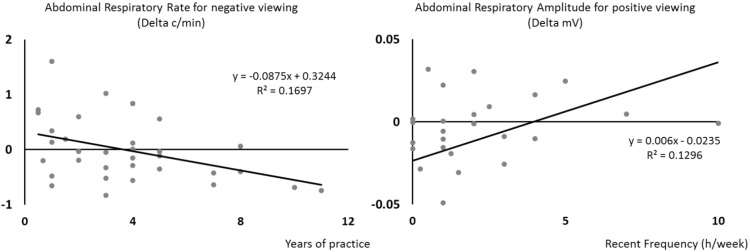
Scatterplots showing that, with an increasing number of years of yoga practice (*x*-axis), participants showed a lower abdominal respiratory rate when seeing negative pictures (*y*-axis, left side), and that, with an increased frequency of recent yoga practice (*x*-axis), participants showed an increasing abdominal respiratory amplitude when seeing positive pictures (*y*-axis). Regression lines, equations, and *R*^2^ for each univariate model are shown.

However, at the abdominal level, two results were significant. The first result shows that with more years of yoga practice, participants showed a lower respiration rate when seeing negative pictures, *F*_(1,31)_ = 6.36, *p* = 0.017, *R*^2^= –0.170, β = –0.41 (**Figure 3**, left side). The second result shows that recent frequency of yoga practice was significantly associated with abdominal respiratory amplitude increase during positive viewing, *F*_(1,30)_ = 5.67, *p* = 0.024, *R*^2^= 0.159, β = 0.40 (**Figure 3**, right side).

## Discussion

In the present study, we investigated the link between yoga practice (years and frequency of practice) and emotion reactivity in healthy populations. We also asked about the reasons individuals practice yoga. To start with, the study was motivated by reports that yoga is a potent means to enhance health and wellbeing or counteract psychopathology. Such positive impacts might result from yoga helping individuals to better handle their emotions, which in itself is a key ability to promote health (Gross and Munoz, 1995; Smyth and Arigo, 2009; Hopp et al., 2011; DeSteno et al., 2013; Hu et al., 2014). To test that yoga practice *itself* links to these benefits, we assessed emotion reactivity in the laboratory in yoga practitioners of increasing experience. We analyzed their self-reported emotional experience and physiological reactions (heart rate, skin conductance, and respiration) to positive and negative emotional material as a function of their reasons to perform yoga (physical and mental), the number of years they practiced, as well as their general and recent frequency of yoga practice.

We found that recent frequency and overall number of years of yoga practice impacted measures of emotional reactivity and so did reasons for practicing yoga, particularly when related to mental reasons. For self-report ratings, we observed that the more people practiced yoga over the last weeks, the less they rated negative pictures as negative and positive pictures as positive. For heart rate, we observed that increasing heart rates when watching positive pictures were associated with a higher tendency to practice yoga for mental reasons. For respiration, we observed that the longer people have experience in practicing yoga (in years), the slower was their respiratory rate in the abdominal region when seeing negative pictures. We also found that the more participants performed yoga in the last month, the deeper was their breathing in the abdominal region when seeing positive pictures. These results should be considered within a methodological framework in which we did not manipulate yoga practice (e.g., using naïve participants, giving them yoga practice and assessing their emotion reactivity before and after). Instead, we followed a more “natural” design in which we recruited participants who themselves had initiated yoga practice in the past. We asked about their experience, recent practice and reasons to practice yoga.

Before discussing our major findings, we would like to reiterate our reasons to use a quasi-experimental design. We worked on the premises that self-initiated yoga practitioners are not directly comparable to persons who start yoga within a psychological intervention context (Villemure et al., 2015). In the case of self-initiation, some individuals might have realized that yoga helps them physically for posture and pain control (Sherman et al., 2005; Cramer et al., 2013a, b). Others might practice yoga for psychological stress control (van der Kolk et al., 2014; Fang and Li, 2015) or spiritual-religious reasons (Macdonald and Friedman, 2009; Varambally and Gangadhar, 2012). In any case, these individuals have initiated their practice on their own terms, based on personal interest and motivation, and have maintained their practice for a varying amount of time (in our study from under 1 year to up to 11 years). As already argued in the introduction, such a complex, mind-body centered practice is unlikely acquired and consolidated in short-term intervention programs of several weeks. Surely, we can expect to observe short-term training effects (Cusumano and Robinson, 1993; Tekur et al., 2008), but would also expect that long-term consolidation effects are differently expressed (Chaya et al., 2006). These considerations were important to us when designing the present study and are also important when discussing our results.

The finding on heart rate indicated that the more people practice yoga for mental reasons, the stronger was their heart rate increase when seeing positive pictures. We found no comparable results on the reasons for practice involving our emotionality ratings or remaining physiological measures (breathing, skin conductance). We propose that interoceptive awareness could have contributed to our finding. In a previous study, individuals high in interoceptive awareness showed a pronounced increase in heart rate when viewing positive as well as negative pictures (Pollatos et al., 2007). Their viewing context was highly similar to the one we used in the present study. In our case, performing yoga for mental reasons might be associated with enhanced interoceptive awareness, leading in turn to heart rate increases when seeing emotional pictures. Yoga-related practices such as mindfulness indicate that this practice helps control one’s heart rate (Delizonna et al., 2009). Particularly high interoceptive awareness might (i) have triggered our increase in heart rate in people specifically performing yoga for mental reasons, or (ii) have motivated further yoga practice to better control heart rate in these individuals. At this point, we report on our findings and propose some explanations. Future studies are needed to support our conjecture and would have to explain why our result was only found for positive pictures.

Control mechanisms might also be key to our second major finding on breathing. For respiratory rate, a slower rate in the abdominal region when seeing negative pictures was associated with a longer experience in yoga practice. In addition, a deeper respiration in the abdominal region when seeing positive pictures was associated with more frequent yoga practice over the last month. Breathing (pranayama) is key to yoga as is meditation and physical practice (asana). There are multiple types of yoga breathing; alternate nostril breathing, retention breathing, ujayi, and localized breathing which consists of breathing in the low belly and chest as distinct or common areas (see for instance Brown and Gerbarg, 2009). Studies showed that experienced yogis shift their breathing in favor of abdominal breathing (Arambula et al., 2001). This shift toward the abdominal region likely demonstrates emotion regulation mechanisms, mainly to support relaxation (see e.g., Dudley et al., 1964; Boiten et al., 1994). Potentially, such “relaxing” breathing reactions may come into play because of the higher arousal level of emotional situations, in particular when of negative nature (Gomez et al., 2005). In our study, skin conductance reactions were, indeed, stronger for negative than positive stimuli (Lang et al., 1993; Garavan et al., 2001; Grühn and Scheibe, 2008). We suggest that long-term as well as short-term yoga practice might enable favorable emotion regulation capacities leading to a shift toward more relaxing or mindful states (see also Gootjes et al., 2011), with potential different mechanisms for negative and positive situations.

We consider response biases such as desirability effects or self-perception to explain our last major finding. We found that negative pictures were judged as being less negative and positive pictures were judged as less positive by individuals who practiced yoga more frequently in recent weeks. We have not explicitly asked participants about their expectations about and attitudes toward yoga. However, one goal in yoga practice is to attenuate the extent of negative and positive feelings (e.g., Menezes et al., 2015), something yoga instructors regularly favor and teach in their daily practice (Kepner et al., 2014). This desirable tendency would not mean that people should feel nothing, but aim to balance their feelings. In other words, the aim is to learn not to live in emotional extremes of either valenced polarity. This balance is sometimes referred to as “equanimity” (Desbordes et al., 2015). In other words, finding an association between yoga practice and a reduction in the reported experience and, to some extent, in physiological arousal, is consistent with the goal of yoga to increase equanimity. We suggest that, with time, participants gradually shift from controlled short-term effects of yoga to more trained, “automatic” physiological control with long-term practice.

### Limitations and Considerations for Future Studies

As for all empirical studies, one could have done certain things differently. Also, during the course of any study, we learn what to do better and study next. As of now, we would like to discuss age effects, lack of neutral pictures, and skin conductance measures.

Firstly, age interacted with (i) the time period people are practicing yoga (which can be expected since the longer individuals practice yoga, the older they are) and (ii) recent frequency of yoga practice. Age did not interact with general frequency of yoga practice. The pitfall with general frequency is that participants estimated general frequency over potentially very different frequencies of yoga practice during different time periods. Thus, results might differ as a function of current practice and not an estimated average frequency. Dynamic of practice could be an interesting independent variable to test. One could examine, for example, whether a long break without yoga followed by a regained interest and intensive yoga period would link with a different emotion reactivity as compared to steady uninterrupted average frequency of yoga practice.

Secondly, we included a couple of neutral pictures as compensatory stimuli, but did not include neutral situations as a condition *per se*. Consequently, we cannot infer the actual valence of our material in our population. At this point, we do not know whether yoga practitioners consider neutral material to be neutral. Ideally, in the case of equanimity (Desbordes et al., 2015), “neutral” remains “neutral,” because extremes would rather be attenuated in affective load toward neutrality. On the other hand, yoga practitioners profiting from an overall enhanced well-being (Carmody and Baer, 2008; Hartfiel et al., 2011) might be generally more positive and balanced, shifting neutral conditions toward positive conditions (see Leppanen et al., 2004 for a complementary example of depression and shift toward negativity). If so, a neutral state might be more positive in yoga practitioners than non-practitioners making differences between a neutral and positive state less important than between a neutral and negative state.

Thirdly, we would like to comment in more details on our skin conductance results. As mentioned, negative pictures (likely more arousing) triggered a stronger increase in skin conductance than positive pictures (see also Lang et al., 1993; Garavan et al., 2001; Grühn and Scheibe, 2008). We have chosen this measure as it is a classical indicator of emotional arousal. Yet, skin conductance levels were unrelated to yoga practice parameters. Reflecting on our results led us to the interpretation of skin conductance as being also classically viewed as an indicator of preparedness (Ohman and Mineka, 2001) and to indicate task engagement (Pecchinenda, 1996). Since we have no *a priori* reason to believe that yoga practitioners are less attentive to threat or engage less in a given task, this may explain the lack of differences. To see whether skin conductance was simply an inappropriate measure to assess participants’ involvement in the task, future studies should use additional measures capturing sympathetic activities (e.g., cardiac output).

## Opening and Conclusion

We were interested in the emotion reactivity of self-initiated yoga practitioners as a function of their reasons to perform yoga (physical, mental), for how long they already practice yoga, as well as their general and recent frequency of yoga practice. We assessed emotion reactivity to positive and negative pictures via self-report and psychophysiological reactions (heart rate, skin conductance, respiration). We propose that our physiological results demonstrate automatic-entrained effects of yoga practice in the long-term (slower abdominal breathing when facing negative pictures, increasing heart rate for positive picture types with enhanced mental reasons to practice yoga) and short-term (deeper abdominal breathing when facing positive pictures); all effects likely representing emotion regulation strategies. At the same time, we propose that the self-report findings (lower emotionality of positive as well as negative pictures as a function of recent frequency of yoga practice) represent expectation or desirable response tendencies, namely what individuals expect yoga to have as an effect. All in all, the physiological results raise the question whether yoga practice enables shifts in emotion reactivity toward favorable emotion regulation capacities.

Accepting the richness of yoga practice traditions, we suggest that future studies should account more specifically for the role of breathing, meditation, and physical aspects in individuals’ yoga practice (see also Villemure et al., 2015). Also, accepting that not everybody profits from any kind of intervention in the same way, it would be relevant investigating how emotion reactivity measures (as assessed here) differ according to individuals who, for unknown reasons, profit from yoga practice versus individuals who, for unknown reasons, do not profit from yoga practice. An important future study would test yoga practitioners and compare them to a group of participants who had once practiced yoga, but stopped for reasons to be determined. We feel it important trying to tease apart the impact of the practice of such old traditions when performed in a clinical – therapeutic context (the “classical” 8 or 12-week intervention program, e.g., Villemure et al., 2015) to a setting such as ours (individuals have chosen by themselves to initiate training).

In all cases, however, we can assume that yoga practice may be considered as an activity that affects emotional unfolding as soon as it is started and that its effects may evolve with time toward different forms of emotional functioning.

## Ethics Statement

This study was carried out in accordance with the National Human Research Act. All subjects gave written informed consent in accordance with the Declaration of Helsinki. The protocol was approved by the CER-VD.

## Author Contributions

EM, CM, and ED-G designed the study. EM and ED-G programed the experiment. EM and ST collected the data. ED-G and EM analyzed the data. CM, ED-G, NP, and EM wrote the manuscript. NP and ST proofread the text.

## Conflict of Interest Statement

The authors declare that the research was conducted in the absence of any commercial or financial relationships that could be construed as a potential conflict of interest.
